# Validation of Automated White Matter Hyperintensity Segmentation

**DOI:** 10.4061/2011/391783

**Published:** 2011-09-06

**Authors:** Sean D. Smart, Michael J. Firbank, John T. O'Brien

**Affiliations:** Institute for Ageing and Health, Newcastle University, Campus for Ageing and Vitality, Newcastle upon Tyne NE4 5PL, UK

## Abstract

*Introduction*. White matter hyperintensities (WMHs) are a common finding on MRI scans of older people and are associated with vascular disease. We compared 3 methods for automatically segmenting WMHs from MRI scans. *Method*. An operator manually segmented WMHs on MRI images from a 3T scanner. The scans were also segmented in a fully automated fashion by three different programmes. The voxel overlap between manual and automated segmentation was compared. *Results*. Between observer overlap ratio was 63%. Using our previously described in-house software, we had overlap of 62.2%. We investigated the use of a modified version of SPM segmentation; however, this was not successful, with only 14% overlap. *Discussion*. Using our previously reported software, we demonstrated good segmentation of WMHs in a fully automated fashion.

## 1. Introduction

Magnetic resonance imaging (MRI) is now widely used in the diagnosis of diseases by doctors and is particularly useful for scanning images of the brain and detecting cerebrovascular disorders. White matter hyperintensities (WMHs) are a common finding in elderly people which are associated with vascular risk factors and an increased risk of decline in cognitive and motor function [1]. A number of methods have been used to quantify the hyperintensities to correlate to clinical data such as visual ratings, volumetric measuring, and WMHs pattern [2–6].

An investigation with the LADIS study cohort found that volumetric measurement was more sensitive than visual rating to detect differences in WMHs between groups with versus without memory symptoms although both volumetric measurement and visual rating detected differences in WMHs relating to age and gait disturbance [7]. 

Currently, there is no accepted gold standard for a fully automated WMHs segmentation program. The SPM package (http://www.fil.ion.ucl.ac.uk/spm/software/) has a widely used segmentation tool which classes brain tissue into grey, white matter, and CSF, using a combination of image intensity and a priori knowledge regarding distribution of tissue types. The default does not include information about WMHs, and these can be misclassified as grey matter [8]. Adding information regarding the a priori distribution of WMHs may help to improve the segmentation of WMHs in SPM.

 The study aims to investigate the ability of SPM to segment WMHs from (a) T1 weighted and (b) T1 + FLAIR images using a priori information about WMHs distribution. Results will be compared to manual segmentation of WMHs from FLAIR images, an in-house WMHs segmentation program [9], and a different previously reported program [6].

## 2. Materials and Method

### 2.1. Subjects

We used 30 MRI scans of subjects randomly selected from a previously published study [10]. We included 10 older subjects with no evidence of dementia as well as 20 subjects with mild-to-moderate severity dementia. Of these, 16 fulfilled criteria for probable Alzheimer's disease according to NINCDS/ADRDA [11], and 4 cases met criteria for probable dementia with Lewy body according to the consensus criteria [12]. The scans were acquired from a Phillips 3T MRI system (Intera Achieva scanner), using the integrated RF body coil for transmission and signal detection through an 8 channel SENSE head coil. All the participants were aged 60+, and basic demographic information was collected, along with a minimental state examination (MMSE) which is used to screen cognitive function and can indicate if a person shows signs of cognitive impairment (Table 1). The study was approved by the local ethics committee. Images acquired included a T1 weighted volumetric sequence covering the whole brain (MPRAGE, sagittal acquisition, slice thickness 1.2 mm, pixel size 1.15 × 1.15 mm; TR = 9.6 ms; TE 4.6 ms; flip  angle = 8°) and FLAIR images to demonstrate white matter hyperintensities (TR 11000; TE 125; TI 2800 ms, data out as 1.016 × 1.016; 60 slices 2.5 mm). The FLAIR and T1 weighted images were spatially registered together using SPM's “coregister” tool.

### 2.2. White Matter Hyperintensity Segmentation

**Figure 1:**
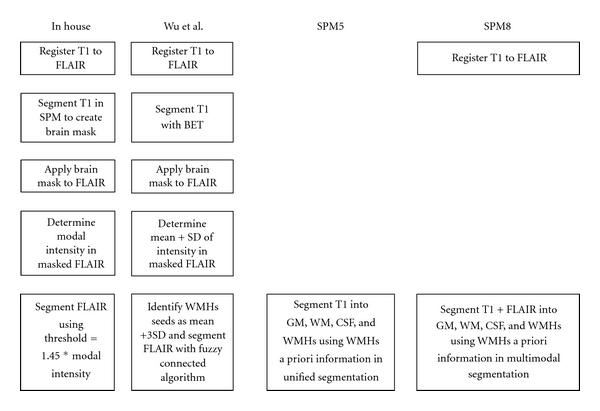
Overview of segmentation procedures.

Figure 1 gives an overview of the steps for each of the automated segmentation processes.

### 2.3. In-House Programme

We have previously described the in-house segmentation routine [9]. Briefly, SPM5 (http://www.fil.ion.ucl.ac.uk/spm/) was used to segment grey matter (GM), white matter (WM), and cerebral spinal fluid (CSF) of the T1-weighted images. A brain mask was then created from GM + WM. The mask was used to remove nonbrain regions from the fluid attenuated inversion recovery (FLAIR) image. The images were then segmented. To perform the segmentation, (a) on the skull stripped FLAIR image, the modal pixel intensity was determined. (b) A threshold-based segmentation was then performed, using a threshold of 1.45 times the modal pixel intensity. (c) Isolated pixels were then removed from the segmentation.

### 2.4. Wu Programme

The programme code was obtained from the author Minjie Wu. This is a brief summary of the process; for more details, see Wu et al. [6]. The programme's automated WMHs segmentation used three main steps; image preprocessing which included (a) coregistering the FLAIR with the T1 image, (b) using the Brain Extraction Tool (part of FSL (http://www.fmrib.ox.ac.uk/fsl/)) on the T1-images to create a brain mask, (c) the brain mask was then applied to the FLAIR images to remove nonbrain tissue. Next, an automated procedure identifies lesion seeds by using an intensity histogram for the image, using the mean plus 3 SD for the minimum threshold and labels these seeds. Afterwards, it uses a fuzzy connected algorithm to segment lesions while iteratively updating the seeds. When the process can no longer detect any seeds, it combines the clusters and is able to produce a mask of WMHs. The code was changed to mean +2.5 standard deviations to see if this would improve the accuracy of the program due to a low overlap with the manual selections with the original code.

### 2.5. Statistical Parametric Mapping (SPM)

Severe WMHs appear as hypointense areas on T1-weighted images, having a similar intensity to grey matter. For this reason, the standard SPM segmentation sometimes misclassifies WMHs as grey matter [8]. The SPM segmentation segments images into a number of separate channels, utilising information regarding the a priori probability distribution of those channels. We investigated the efficacy of including an extra channel for WMHs into the SPM segmentation using an a priori probability distribution of WMHs to improve the accuracy of the segmentation.

### 2.6. Creation of WMHs a Priori Map

We used a different, previously published group of 60 subjects aged over 65 [9] to create an a priori probability distribution of WMHs. FLAIR images from these subjects were segmented using the in-house WMHs segmentation program. The FLAIR images were then affine-transformed into MNI space, using the registration tools in SPM, and the transformation applied to the segmented WMHs. An average of the transformed segmented WMHs images was calculated as the a priori probability distribution of WMHs.

### 2.7. SPM Segmentation

We performed segmentation using both the SPM5 unified segmentation [13] and the multimodal segmentation “new segment” in SPM8. The SPM5 segmentation was performed on the T1 weighted images, whilst the SPM8 was done as a multimodal segmentation using both the T1 and FLAIR images. In both cases, we added an extra segmentation channel, using the a priori probability distribution of WMHs just described.

### 2.8. Manual Segmentation

Manual selection was done using in-house software written in java language by a trained biomedical sciences undergraduate student (SS). Using a mouse, the operator selected WMHs individually and then altered the intensity threshold to best outline the area of the WMHs. The program labelled these as regions of interest (ROI). Accuracy and validation of manual selection was done by self-comparison, two weeks after the original selection, and comparison to another operator with extensive experience of WMHs segmentation (MJF) over a random selection of scans from the cohort. These segmentations were compared to the original masks to find the overlap between the accuracy checks and the original selections.

### 2.9. ROI Overlaps and Quantitative Results

To compare segmentation methods, we calculated the overlap using

(1) $$\text{Overlap}=100\times \frac{\left({\text{ROI}}^{1}\cap {\text{ROI}}^{2}\right)}{\left({\text{ROI}}^{1}\cup {\text{ROI}}^{2}\right)},$$

utilising the *fslmaths* and *fslstats* tools in FSL.

We calculated the overlap of the manual segmentation versus all the automated methods.

## 3. Results

**Figure 2:**
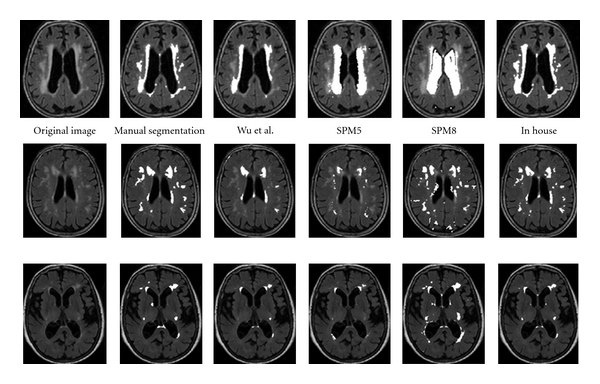
Segmentation results. From left: raw image, manual segmentation, segmentation with Wu et al. software, SPM5 segmentation with WMHs a priori probability included, SPM8 multimodal segmentation with WMHs a priori probability included, and segmentation with in-house software.

The results are summarised in Table 2.  Figure 2 shows examples of typical segmentation.

### 3.1. In-House Program

The in-house program produced an average overlap of 62.24% (S.D. ± 11.45%) with the manually selected regions chosen by the operator similar to the disagreement between the two operators. On visual inspection, causes of disagreement were that the in-house program does tend to misclassify where blood flow can be seen on an image or on brain slices that display bright grey matter. This occurs on mostly on scans that do not have a large volumetric mass of WMHs to begin with and possibly caused by the modal intensity of the whole scan being low. In scans that appear to show large volume of WMHs (over 10,000 mm^3^) the in-house program was very accurate over 70% overlap with the manual selection. These scans had easily definable WMHs to be marked, and the large masses of the lesions usually covered any errors misclassifying tissue.

### 3.2. Wu Program

The Wu program produced a mean overlap of 36.81% (S.D. ± 18%) which is markedly lower than the in-house program and also differs from the original work which appears to show good ability to identify WMHs accurately from other matter. The highest was 83.07%, while the lowest was 11.11%; the actual lowest was 2.19% but this was caused due to the preprocessing stage not properly stripping the skull from the image rather than an error in how the images were segmented. On one scan, there was an abnormally low segmentation of WMHs, on visual inspection the program appeared to be outlining small regions within the WMHs and not the whole WMHs itself. Altering the number of standard deviations to change the minimum threshold did not improve accuracy; instead, the program became more inaccurate as more non-WMHs were included in the segmentation. There was a tendency for deep WMHs and smaller clusters of WMHs to be not included in the segmentation (see Figure 2).

### 3.3. Statistical Parametric Mapping

The SPM5 segmentation using just the T1 weighted images produced a mean overlap of 14.4%. The SPM8 segmentation using both T1 and FLAIR produced an average overlap of 11.9% (S.D. ± 18.5%). 

Visual inspection showed the errors on the segmentation with just the T1 images to be from a variety of causes, both misclassification of non-WMHs regions and underestimation of WMHs. The segmentation worked best when WMHs were large in extent. The presence of large ventricles (which overlapped with the a priori WMHs distribution) or of subtle WMHs reduced the accuracy of the segmentation. There was a tendency for the WMHs segmentation to include regions of CSF (see Figure 2) with the SPM5 segmentation including typically the ventricles, whilst the multimodal SPM8 often included sulcal CSF. The SPM5 software also often missed deep WMHs, which were located in regions of low expectation of WMHs in the a priori map.

We had expected adding the FLAIR to the segmentation to improve the results, however, this was not the case. Visual inspection showed that the SPM8 segmentations using T1 and FLAIR was very nonspecific—while regions of WMH were identified correctly, a large amount of non-WMHs were also misclassified, leading to an overall poor performance. For example, in the middle row of Figure 2, whilst the majority of the WMH have been segmented correctly, there are regions in the ventricles and around the sulci which have been incorrectly segmented as WMHs. In the lower row, the SPM8 segmentation has included a much larger region of white matter than the manual segmentation.

## 4. Discussion

Out of all the programs, the in-house program produced the most favourable results as a fully automated method to identify WMHs. Although only producing a reasonable accuracy for the majority of the scans, it is currently only reliable to give sufficiently accurate measurements on scans with large masses of WMHs visible. A possible method to improve accuracy would be to provide a template that can mask arteries within the brain to prevent segmentation in the area because blood flow appears as bright pixels, brighter than the WMHs, and is often selected in the segmentation process. Wu et al. reported a high success in accurately identifying WMHs in the original work [6]; however, we were unable to replicate this. Their segmentation used the SD of histogram to determine the threshold for segmenting WMHs and possibly differences in SNR between scanners affected results. Our in-house segmentation seems relatively robust to scanner differences, as the original development and testing was done on a 1.5T scanner, and it still produced a reasonable accuracy on the images from the Phillips 3T scanner. The SPM programs could segment large easily defined WMHs masses in images with reasonable accuracy (approx 67%); however, the segmentations were poor in other cases. For SPM5, the segmentation uses T1-weighted images for segmentation, and WMHs may not be as clear on these images, as they are on FLAIR images although SPM8 segmented using a dual channel of T1-weighted and FLAIR images and was on average less accurate. The dual channel approach was nonspecific, and although regions of WMHs were correctly identified, large regions of other tissue were misclassified as WMHs. One difficulty with segmenting the WMHs with the SPM approach is that WMHs are not always present, unlike GM and WM which are always present in a brain, albeit in subject dependent morphology. Hence an FLAIR image of a subject with no WMHs has a very different intensity distribution to that of a subject with a large volume of WMHs. Including information regarding the likely local spatial and intensity characteristics of WMHs may help improve the segmentation.

We confirmed the accuracy of our in-house segmentation code on 3T images, with 70% overlap against manual segmentation. The code is suitable for large-cohort investigations of WMHs in aging.

## Figures and Tables

**Table 1:** Subject demographic details. WMHs volume is from manual segmentation.

	Control	AD	DLB
Age: mean (SD)	77.2 (9.0)	77.3 (8.9)	75.5 (5)
Sex F : M	2 : 8	8 : 8	2 : 2
MMSE mean (range)	28.9 (27–30)	21.1 (16–27)	17.8 (15–22)
Hypertension *N* (%)	4 (40%)	6 (33%)	1 (25%)
WMHs volume median mL (range)	4.0 (0.8–46)	4.2 (1.0–34)	5.4 (1.9–28)

**Table 2:** Percent overlap of segmentation methods against manual segmentation.

	Mean (SD)	Range
Within rater repeat (*N* = 5)	71.4 (12)	
Between rater repeat (*N* = 5)	63.3 (18)	
In-house segmentation	62.2 (12)	38–85
Wu segmentation	36.8 (18)	11–83
SPM5 T1 segmentation	14.4 (21)	1–69
SPM8	11.9 (19)	1–65
